# Direct visualization by FRET-FLIM of a putative mechanosome complex involving Src, Pyk2 and MBD2 in living MLO-Y4 cells

**DOI:** 10.1371/journal.pone.0261660

**Published:** 2021-12-23

**Authors:** Richard N. Day, Kathleen H. Day, Fredrick M. Pavalko

**Affiliations:** Department of Anatomy, Cell Biology and Physiology, Indiana University School of Medicine, Indianapolis, Indiana, United States of America; University of Virginia School of Medicine, UNITED STATES

## Abstract

Earlier, we proposed the “mechanosome” concept as a testable model for understanding how mechanical stimuli detected by cell surface adhesion molecules are transmitted to modulate gene expression inside cells. Here, for the first time we document a putative mechanosome involving Src, Pyk2 and MBD2 in MLO-Y4 osteocytes with high spatial resolution using FRET-FLIM. Src-Pyk2 complexes were concentrated at the periphery of focal adhesions and the peri-nuclear region. Pyk2-MBD2 complexes were located primarily in the nucleus and peri-nuclear region. Lifetime measurements indicated that Src and MBD2 did not interact directly. Finally, mechanical stimulation by fluid flow induced apparent accumulation of Src-Pyk2 protein complexes in the peri-nuclear/nuclear region, consistent with the proposed behavior of a mechanosome in response to a mechanical stimulus.

## Introduction

Cell biology research using animals, plants and bacteria have revealed many examples in which the biologic activity of cells and tissues is altered by mechanical stimulation from the cell’s external environment (a process collectively known as cellular mechanotransduction). One such example in which cellular mechanotransduction is particularly important to human health is seen in the mammalian skeleton. The application of external mechanical loads (such as occur during exercise, walking and even basic everyday movements) stimulate bone remodeling, increases net bone formation and contributes to the maintenance of a healthy skeleton throughout life. The health benefits of maintaining a strong and adaptable skeleton is particularly important in humans where physical activities drive life-long adaptation of the skeleton to the mechanical demands placed upon it [1]. The negative consequences of defective skeletal mechanotransduction are seen in many human conditions. These include common skeletal diseases like osteoporosis, patients unable to move their limbs, astronauts exposed to long periods of microgravity in space, and, of great consequence to an aging world population, what we now accept as the normal loss of bone mass during aging (particularly severe in estrogen-deficient, post-menopausal women). A better understanding the molecular mechanisms that mediate cellular mechanotransduction and how load-induced bone remodeling can become dysfunctional has important consequences to human health.

In this study we sought to better understand the molecules involved and the sub-cellular mechanisms that mediate mechanotransduction in the most abundant of all types of bone cell, the osteocyte [2]. Understanding mechanotransduction mechanisms is broadly relevant to cell biology. In bone cells specifically this work can aid efforts to develop effective counter measures to disease and age-related bone loss. Here, we describe efforts to directly visualize the physical interaction between the constituent proteins in a “mechanosome” an intracellular structure first proposed in 2003 [3] to translate mechanical stimuli detected by mechanosensors at the cell surface into biochemical signals that alter gene expression.

Theoretical multi-protein complexes termed “mechanosomes” [3] were proposed to function within cells as vehicles to transmit mechanical signals detected at the cell surface into the cytoplasm, peri-nuclear regions and nucleus to modulate cell biochemistry and gene expression. Mechanosomes were predicted to be “anchored” to the cytoplasmic domains of certain membrane proteins called “mechanosensors” that are capable of sensing mechanical stimuli. When mechanically stimulated (by forces such as substrate strain, membrane stretch or fluid shear stress), mechanosensors would undergo conformational changes that release mechanosomes from their attachments to the mechanosensor’s cytoplasmic domain, thus “launching” mechanosomes into the cell interior. Fundamentally, mechanosomes might function analogously to other signal transduction complexes that are activated in response to a cytokine or growth factor binding to its receptor. The difference being that mechanosensors respond to mechanical stimuli not receptor ligands and signal to mechanosomes to translocate into the cytoplasm and toward the peri-nuclear region and possibly enter the nucleus to alter cell biochemistry and gene expression.

Two membrane structures known to function as mechanosensors are cadherin/β-catenin/LRP5 complexes (cell-cell anchoring junctions) and integrin/focal adhesion complexes (adhesive junctions between cells and the extracellular matrix) [3–5]. Until now, however, direct demonstration or visualization of multi-protein mechanosomes in living cells has not been possible. Here, we directly visualize a multi-protein mechanosome complex in focal adhesions, the cytoplasm and peri-nuclear/nucleus of living cells. This mechanosome appears capable of responding to an applied fluid shear stress by translocating from the cell membrane (focal adhesions) and accumulating toward the cell interior where it interacts with MBD2 (Methyl-CpG-binding domain protein-2) to potentially alter expression of genes in response to mechanical stimulation.

The mechanosome described in this study consists of at least three proteins: Src (sarcoma tyrosine kinase, Pyk2 (Proline-rich kinase-2), and MBD2, which together have been proposed to be capable of functioning as a physical communication link (a mechanosome) between mechanical signals detected at the osteocyte cell membrane (via integrins in focal adhesions) and the osteocyte nucleus and peri-nuclear region surrounding the nucleus [6]. Mechanosomes in bone cells may regulate expression of mechanically sensitive skeletal proteins, such as the Sost gene product sclerostin, just one of many well-characterized regulators of load-induced bone formation that function in the Wnt-β-catenin signaling pathway [7–12].

Src and Pyk2 are tyrosine kinases that associate with the cytoplasmic domain of integrins in focal adhesions, sites of interaction between cells and the extracellular matrix (ECM) [13]. Focal adhesions have been proposed as likely sites of mechanosensation in cells, being perfectly positioned at sites of cell-ECM interaction to detect changes in fluid shear stress or substrate strain [14–17]. MBD2 is a methylated DNA binding protein that is localized primarily in the nucleus [6, 18, 19]. We reported previously [6] that subjecting osteoblasts to 1 hour of fluid flow caused increased accumulation of Src and Pyk2 in the peri-nuclear matrix that surrounds the nucleus using indirect immunofluorescence microscopy. We also previously demonstrated increased Src kinase activity in the nucleus of osteoblasts following 10–20 minutes of fluid flow (detected using a biosensor that measured Src kinase activity, [20]). Using co-immunoprecipitation from osteoblast lysates, Pyk2 and MBD2 formed a complex that could potentially also interact with phosphorylated Src (Y418) after osteoblasts were subjected to 20 minutes of fluid flow [6]. Together, these previous results using antibody-based approaches in fixed and lysed cells suggested that a Src, Pyk2 and MBD2 multi-protein complex could function as a mechanosome that contained necessary elements for translating a mechanical stimulus (fluid flow) into changes in expression of genes in the nucleus. However, direct evidence supporting the existence of a Src-Pyk2-MBD2 mechanosome, and a potential role of this complex in living cells in response to mechanical signals such as fluid flow from the cell surface to the peri-nuclear matrix and nucleus required new molecular tools.

Here we report the creation of FP (fluorescent protein)-tagged Src, Pyk2 and MBD2 protein constructs suitable for co-expression in living MLO-Y4 osteocyte cells that have allowed us to visualize and quantify a mechanosome complex by FRET-FLIM (Förster resonance energy transfer-fluorescence lifetime imaging microscopy) in living cells. FLIM is used to quantify the excited-state lifetime of fluorescent proteins in different cellular environments. The fluorescence lifetime is an intrinsic property of every fluorophore, and it carries information about events that affect the excited-state. FRET is an excited-state process that quenches the donor fluorescence and shortens the donor lifetime. Since FLIM can quantify this change in lifetime, it is a powerful method to measure FRET between proteins inside the living cell [21]. Importantly, because the measurements are made in based on time, not just fluorescence intensity, they are unaffected by variations in probe concentration, changes in the excitation intensity, and, provided there is sufficient signal-to-noise, will be minimally affected by light scattering. We used FRET-FLIM to obtain detailed sub-cellular information on the distribution of this complex in living cells and suggest the possibility that the formation and intracellular movement of a mechanosome complex may be altered by the application of fluid shear stress.

## Materials and methods

### FRET standards and fusion protein constructs

The FRET standards were produced using either monomeric (m)Cerulean3 (mCer3) [22], mVenus [23], or mScarlet [24]. The donor fluorophores (mCer3 or mVenus) were coupled directly to mScarlet through a 10 amino acid (aa) linker as described earlier [25]. Cerulean3 is based on *Aequorea* GFP Cyan variant Cerulean, and Scarlet is based on mCherry that was derived from DsRed from the coral *Discosoma striata*. The FRET standards were used to verify the FRET-FLIM measurements. The vectors for FP-tagged proteins Src, MBD2 and Pyk2 are based on the Clontech C1 and N1 plasmids, and fusion protein expression is driven by the CMV promotor. The linker between Src and Scarlet (Scarlet is located at the C-terminus of the fusion protein) and between Src and Venus (Venus is located at the N-terminus of the fusion protein) is “SDPT”, the linker between Cer3 and mPyk2 and between Cer3 and Src is “SGLRSRAQASNSAVDGTAGPG” (Cer3 is located at the C-terminus of the fusion protein). The linker between MBD2 and Venus and Pyk2 and Venus (Venus is located at the N-terminus of the fusion protein) is “FGSRG”.

For the Cer3-Pyk2 construct, mouse Pyk2 cDNA was kindly provided by Dr. Tere Bellido, Indiana University. To create Cer3-Src and Scarlet-Src, mouse Src cDNA was obtained from Addgene (#13663, Watertown, MA). Cer3 and Scarlet cDNA was obtained from Addgene (#85044 & #98839, respectively). To create Venus-MBD2, mouse MBD2 cDNA was obtained from ATCC. All plasmid inserts were confirmed by direct sequencing.

MLO-Y4 osteocytes were obtained from Dr. Lynda Bonewald, Indiana Center for Musculoskeletal Health, Indiana University School of Medicine, Indianapolis, IN, and were cultured on collagen-coated plates (rat tail collagen type I, BD Biosciences, San Jose, CA) in MEM-α (Gibco, Life Technologies, Carlsbad, CA) supplemented with 5% calf serum, 5% fetal bovine serum (FBS) and 1% penicillin/streptomycin (Gibco, Life Technologies, Grand Island, NY). Cells were maintained in 5% CO2 at 37° C and used for imaging experiments when they were approximately 60–70% confluent. One day prior to imaging, MLO-Y4 cells were electroporated at 250 volts with a 9 ms pulse duration using a BTX ECM 830 electroporator (Harvard Apparatus, Holliston, MA) as described earlier [20]. The cells are immediately recovered from the cuvette and diluted in phenol red-free MEM containing 10% fetal calf serum. The suspension is transferred to sterile two or four well-chambered cover glass (Lab-Tek II, Thermo Scientific, Waltham, MA), which are placed in an incubator (37°C and 5% CO_2_-Air 95%) prior to FLIM the following day.

### Cell imaging

MLO-Y4 cells were transfected by electroporation, in triplicate with plasmids encoding either Src-Venus, MBD2-Venus or Pyk2-Venus and transferred to individual wells of 6-well culture plates. Non-transfected cells were grown in a separate well and the plates were placed in an incubator (37°C and 5% CO2-Air 95%) prior to imaging and recovery of lysates for Western blotting the following day. The cells were imaged for brightfield and Venus FP fluorescence using an Olympus IX71 microscope equipped with a 10-X air objective. The fluorescence images were acquired with a Semrock YFP-2427A filter and captured with a Hamamatsu Orca R2 CCD (charge-coupled device) camera using Micromanager and ImageJ software. For overlay, the fluorescence image was converted to indexed image and a lookup-table from red to white was applied using Vistavision software (ISS Inc., Champagne, IL). This image was then merged with the corresponding bright-field image.

### Western blot analysis

**Fig 1:**
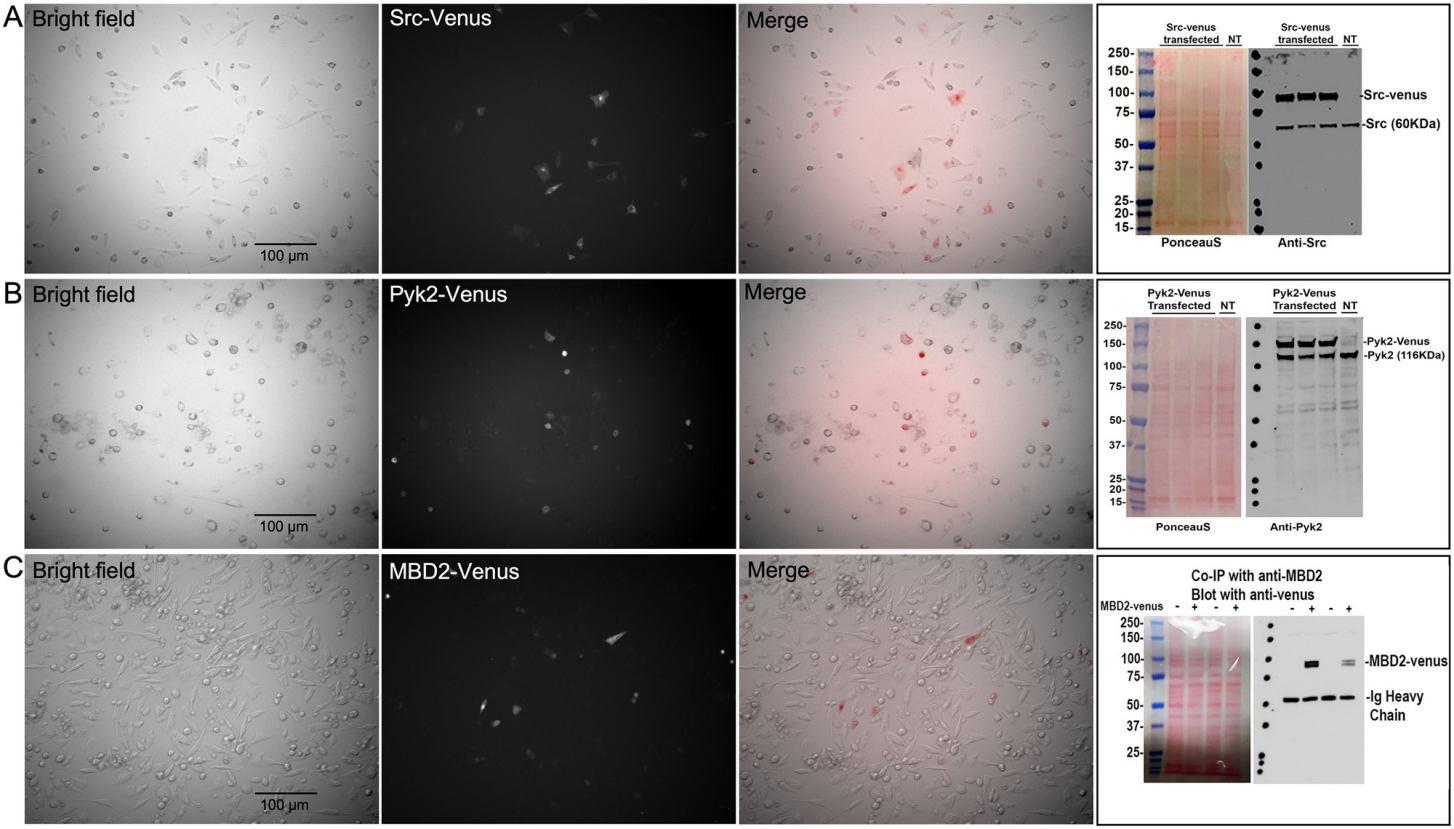
Evaluation of fluorescent protein expression levels in MLO-Y4 cells. Rows A, B and C show cells transfected with either Src-Venus, Pyk2-Venus or MBD2-Venus respectively, and imaged at low (10X objective) magnification to evaluate the range of expression levels across a population of cells. In each row, a representative Brightfield image is shown (to show all cells), the corresponding fluorescent channel (to show transfected cells expressing the fusion protein) and a merged image of Brightfield and fluorescence. For Src-Venus and Pyk2-Venus cells were also harvested (in triplicate) and analyzed by immunoblot to estimate the relative levels of exogenous (FP-tagged) and endogenous protein using antibodies against Src (Row A) or Pyk2 (Row B). Antibodies against Src and Pyk2 recognized both the endogenous protein as indicated as well as the higher molecular weight Venus-tagged fusion protein (migrating approximately 27 KDa higher than the endogenous protein, consistent with the expected size of Venus; Rows A and B). Densitometry of bands in all three replicates for both Src and Pyk2 showed that the Venus-tagged fusion protein were expressed at levels between 2–4 times higher than the endogenous protein. In contrast, MBD2-Venus fusion protein could not be detected at the predicted molecular weight (~90–95 kDa) by any of 4 different antibodies against MBD2 that were able to recognize endogenous MBD2 (not shown). We concluded that MBD2-Venus was expressed at much lower levels than endogenous MBD2. As an alternative approach to confirm expression of MBD2-Venus, we performed a co-immunoprecipitation (co-IP) experiment using antibody against MBD2 for the IP, following by an anti-GFP antibody for the western blot of co-IP complexes (Row C). Antibody against GFP (which recognized Venus) detected a band at a molecular weight of approximately 90–95 KDa corresponding to the expected size of the MBD2-Venus FP.

Cells extracts for western blotting were obtained from MLO-Y4 cells grown in 60 mm dishes with or without plasmid transfection of FP-tagged protein. For each trial triplicate wells transfected with plasmid expressing FP-tagged protein were collected and a single well of non-transfected cells (NT, in Fig 1) were collected. Cells were washed 3X with PBS to remove serum proteins before whole cell extracts were collected directly in 150 uL of 2X SDS-PAGE sample buffer (0.15 M Tric-HCl, pH 6.8, 1% DTT, 1% SDS, 30% glycerol, 0.05% bromophenol blue) and heated to 95 C for 5 min. DNA in the sample was sheared by repeated passage through a 25g needle (to reduce viscosity of the sample) and approximately 20 ug of sample per lane was loaded onto 4–12% gradient polyacrylamide gels (GenScript, Piscataway, NJ, USA). Proteins were separated by SDS-PAGE electrophoresis along with a separate lane of pre-stained molecular weight markers (Bio-Rad). Separated proteins were transferred to nitrocellulose overnight at constant 25 Volts, after which the membranes were rinsed briefly in TBST (Tris-buffered saline, 0.1% tween 20) wash buffer, stained with PonceauS to visualize total protein on the nitrocellulose and qualitatively evaluated for protein loading consistency. The nitrocellulose was de-stained by brief washes with TBST and then excess protein-binding sites were blocked by incubation with 5% powdered milk dissolved in TBST for 1 hour. The nitrocellulose was then incubated with primary antibodies (diluted in 5% milk/TBST) at 4°C overnight, washed 6× for 5 min each in TBST, then incubated with species-appropriate HRP-conjugated secondary antibody for 1 hour at room temperature. After 6X washes of 5 min each in TBST, the nitrocellulose was rinsed in deionized water and incubated in ECL (enhanced chemiluminescence) reagent to detect HRP for 5 minutes (ECL Prime Reagent, Amersham, Little Chalfont, UK). Nitrocellulose blots were imaged using an iBrightCL1000 imaging system (Invitrogen, Carlsbad, CA, USA) and analyzed using ImageJ.

### Antibodies used

Antibodies used were: rabbit monoclonal anti-Src (#32G6, Cell Signaling Technologies, Danvers, MA) diluted 1:5000; rabbit polyclonal anti-Pyk2 (#3292, Cell Signaling Technologies, Danvers, MA) diluted 1:2000; Rabbit polyclonal anti-MBD2 (#ab224827, Abcam, Cambridge, MA diluted 1:1000; 3 additional MBD2 antibodies were used in (unsuccessful) efforts to detect FP-MBD2 including: rabbit anti-MBD2 (#NB100-81657, Novus Biologicals, Centennial, CO); mouse monoclonal anti-MBD2 (#N2C3, GeneTex, Irvine, CA); and rabbit polyclonal anti-MBD2 (#ab38646, Abcam, Cambridge, MA). GFP was detected using mouse monoclonal anti-GFP (Living Colors GFP monoclonal antibody, Clontech, Mountain View, CA) diluted 1:10,000; HRP-goat-anti-mouse IgG antibody or HRP rabbit-anti-mouse IgG (Jackson ImmunoResearch, West Grove, PA, USA) diluted 1:10,000. Protein A-sepharose was used to precipitate immune complexes in immunoprecipitation experiments.

### FLIM measurements

The fluorescence lifetime measurements are made using the ISS Alba 5 FastFLIM system (ISS Inc., Champagne, IL) coupled to an Olympus IX71 microscope equipped with a 60-X, 1.2 numerical aperture water-immersion objective lens. A Pathology Devices (Pathology Devices, San Diego, CA) stage-top environmental control system maintains the temperature at 37°C and 5% CO_2_-95% Air. The two-channel Alba laser scanning confocal system is controlled by ISS VistaVision software, which allows independent adjustment of the pinholes, and positioning of the detectors for both channels. The system is equipped with a 448 nm diode laser and a Supercontinuum white light laser (WLL). For the WLL, wavelengths below 690 nm are passed to a filter wheel with excitation filters for green (510/10 nm), or red (561/14 nm). The system uses a 445/515/561 triple-notch filter (Semrock, Rochester, NY) for dual laser excitation. The maximum laser power measured at the specimen plane for the 448 nm and 510 nm lasers was 130 μW and 80 μW, respectively. The typical laser power used for these experiments was in the range of 200 nW.

For FLIM the 448 nm diode laser and WLL are both modulated at a fundamental frequency of 20 MHz, with additional measurements at up to 8 sinusoidal harmonics (20–180 MHz). Both lasers are delivered by a single-mode polarization-maintained optical fiber to the confocal scanning system, which is controlled by the VistaVision software (4.2, Build 163, ISS Inc., Champagne, IL). For 448 nm excitation (mCer3) the fluorescence signals emitted from the specimen are routed by a 495 nm long pass beam splitter through 474/23 nm (cyan emission) or either a 543/22 nm (green emission) or 609/54 nm (red emission) band-pass emission filters. For WLL 510 nm excitation (mVenus) the fluorescence signals emitted from the specimen are routed by a 568 nm long pass beam splitter to 543/22 nm (green emission) or 609/54 nm (red emission) band-pass emission filters. The signals are detected using two identical avalanche photodiodes (APD, SPCM-AQRH-15, Excelitas Technologies, Quebec, Canada) that have an output pulse width of 10 ns, and a transient time spread (TTS or jitter) for single photon time resolution is 350 ps at 825 nm (FWHM). The software records the time resolved photons in different cross frequency phase bins to generate the phase histogram. The information at higher harmonics is obtained by the digital frequency transform of the phase histogram. The phase delays and modulation ratios of the emission signal are measured at each pixel of an image for each frequency. The cyan channel is calibrated with Coumarin6 (Millipore Sigma, St. Louis, MO) dissolved in ethanol (2.5 ns lifetime, [26]) and the green channel is calibrated with 5 μM solution of fluorescein (Millipore Sigma, St. Louis, MO) dissolved in 1 mM NaOH (4 ns lifetime, [27]). The red channel is used for imaging only, but was calibrated for lifetime measurements using Alexa 568 (3.6 ns lifetime, [28]).

### Orbital rotating platform fluid flow conditions

MLO-Y4 cells were plated into individual wells of 6 well plates at ~1.2 x 10^5^ cells per well (~1.1 x10^5^ cells/ cm^2^) in MEM supplemented with 5% calf serum and 5% FBS and antibiotics. Cells were subjected to dynamic fluid flow generated by 2 ml of media on an orbital platform shaker rotating at a speed of ~200 rpm (2Hz) for 1 hour inside a tissue culture incubator at 37°C with 5% CO_2_. Static controls plates were held at 37°C with effort to minimize fluid movement.

### Statistical analysis

Statistical significance was assessed by Kruskal-Wallis one-way analysis of variance (ANOVA) on Ranks followed by pairwise multiple comparison (Dunn’s), or by unpaired t-test with a p-value of p<0.05 or less interpreted as statistically significant. All experiments were repeated 2–4 times and at least 10 cells from each condition were collected in each experiment. Multiple ROI were analyzed from each cell and that number is stated in the text, figure or figure legend. FRET efficiency was determined by: $E_{FRET}=1-\left(\frac{\tau_{DA\;}}{\tau_{D}}\right)$, where E_FRET_ is the estimated efficiency of fluorescence energy transfer, τDA is the fluorescence intensity of the donor fluorophore with acceptor present and τD is the fluorescence intensity of the donor fluorophore without any acceptor present.

## Results

### Characterization of fluorescent proteins

The goal of this study was to use live-cell imaging of an immortalized osteocyte cell line, MLO-Y4 cells to characterize the potential interactions between the putative mechanosome proteins Src, Pyk2 and MBD2. These cells were chosen based on our long-standing interest in determining mechanisms of mechanotransduction at the cellular level in bone, particularly using osteocyte cell models. MLO-Y4 cells are an immortalized cell line derived from mouse osteocytes and are well characterized and widely used in studies of osteocyte function, including studies on osteocyte mechanotransduction [29, 30]. Here, the mCer3, mVenus and mScarlet were used as genetically encoded fluorescent tags for the mechanosome proteins. To demonstrate that Cer3 could serve as a FRET donor for either mVenus or mScarlet, and to show that mVenus could serve as a FRET donor for mScarlet, we constructed FRET standards containing these FPs. In S1 Fig, cells expressing either the mCer3-10AA (Amino Acid)-mVenus, mCer3-10AA-Scarlet, or mVenus-10AA-mScarlet fusion proteins were imaged using FLIM and the lifetime of the donor fluorophores was measured, allowing us to quantify the FRET efficiency (E_FRET_) for each of the standards. The results show the expected quenching of the donor lifetime consistent with energy transfer for each of the FRET standards when compared to the donor alone. Together, these results demonstrate that these FP tags are suitable for use in our FRET-FLIM experiments.

To determine the sub-cellular distribution of the FP-tagged mechanosome proteins Src, Pyk2 and MBD2, and to assess their suitability for these studies, we transfected each FP-tagged construct individually into MLO-Y4 cells (shown in S2 Fig). S2 Fig shows three representative examples each of the distribution of MBD2-Venus, Pyk2-Cer3 or Src-Cer3 expressed in living MLO-Y4 cells. The distribution of the expressed FP-MBD2 (primarily nuclear), FP-Src and FP-Pyk2 (primarily in focal adhesions, in the peri-nuclear region and to a lesser extend in the nucleus) reflects the distributions widely reported in the literature using indirect immunofluorescence [31–36]. Although indirect immunofluorescence studies are limited by cross-reaction of antibodies with off-target cellular proteins and other non-specific background staining, the cellular location of the individual endogenous proteins and the distribution of expressed FP-MBD2, FP-Src and FP-Pyk2 in S2 Fig are consistent.

In order to minimize the chances of adversely affecting the normal biology of transfected proteins in studies relying on protein over-expression it is important that the exogenous protein not be expressed at levels that are substantially above that of the endogenous protein. To determine the relative level of expression of FP-tagged protein compared to endogenous protein, we next transfected MLO-Y4 cells with each of our FP-tagged constructs (single construct transfections) and performed immunoblot analysis of cell lysates in triplicate to estimate the relative level of FP- and endogenous protein expression (Fig 1). We also imaged these cells at low (10X) magnification to estimate the range of expression seen among the population of cells (Fig 1). FP-Src and FP-Pyk2 constructs appear to be overexpressed on average approximately 2-4-fold relative to endogenous protein based on densitometry of bands by western blot analysis of transfected cells. The FP-Src to endogenous Src ratio for each of the n = 3 replicates were 2.9-, 3.0- and 3.3-fold. The FP-Pyk2 to endogenous Pyk2 ratio for each of the n = 3 replicates were 2.4-, 3.6 and 3.1 fold. Both the endogenous and the expressed FP-tagged protein were detected on the same blots using antibodies against either Src or Pyk2, with the FP-tagged protein migrating at a size approximately 27 KDa (kilodalton) higher than the endogenous protein as expected (Fig 1A and 1B). While we cannot know precisely how this level of FP-Src and FP-Pyk2 overexpression affects the interaction between FP-tagged and endogenous cellular proteins, or how this level of expression affects the normal biological activity between endogenous proteins, we were able to confirm that our FP constructs are not dramatically overexpressed relative to endogenous protein. It should be noted however that FP overexpression might be significantly higher than these average values since not all cells are expressing FPs. In our experience, this level of overexpression falls within a range that is typical for similar studies that utilize expressed tagged proteins as a proxy for the behavior of the endogenous protein and had no apparent effect of cell morphology.

Unlike FP-Src and FP-Pyk2, FP-MBD2 expression appeared to be very low relative to endogenous MBD2. If fact, we were never able to detect FP-MBD2 by western blot using antibodies that recognize endogenous MBD2. We tried 4 different MBD2 antibodies that were able to recognize the endogenous protein but none of these antibodies also detected a higher molecular weight band corresponding to FP-MBD2 in transfected cells. This suggested that FP-MBD2 is expressed at levels that are considerably lower than endogenous MBD2. We were, however, able to confirm that FP-MBD2 was expressed by co-immunoprecipitation using an antibody against MBD2, followed by blotting with an anti-GFP antibody that detected the FP (Venus) tag at a molecular weight consistent with the predicted size of MBD2-Venus (Fig 1C). But of course we cannot directly compare expression levels of FP-MBD2 and endogenous MBD2 using this approach. Low magnification fluorescent images of FP-tagged MBD2 protein also confirmed expression of the fusion protein. The proportion of cells expressing FP-MBD2 was not obviously different from the proportion expression FP-Src and FP-Pyk2 indicating comparable transfection efficiency among the FP constructs, therefore we suspect that the level of expression in FP-MBD2 transfected cells is simply lower than for the other constructs. As expected, and as is typical in studies using transfected FP-tagged constructs, we observed a wide range of expression levels among individual cells within the population of transfected cells. For all three FP constructs, approximately 20–30% of cells did not appear to express detectable FP-protein. The majority of cells did express detectable FP-protein, with approximately 10–20% of single-transfected cells expressing levels suitable for FRET-FLIM imaging. Because our experimental FRET-FLIM experiments required us to select those transfected cells that co-expressed either two or three FP-tagged proteins, we carefully selected for analysis only cells that had a morphology that is typical of MLO-Y4 cells and avoided cells with very high levels of FP-protein expression. Additionally, it is important to note that an advantage of the FLIM approach is that lifetimes are independent of the concentration of the donor fluorophore.

### Pyk2-Src FRET-FLIM studies

**Fig 2:**
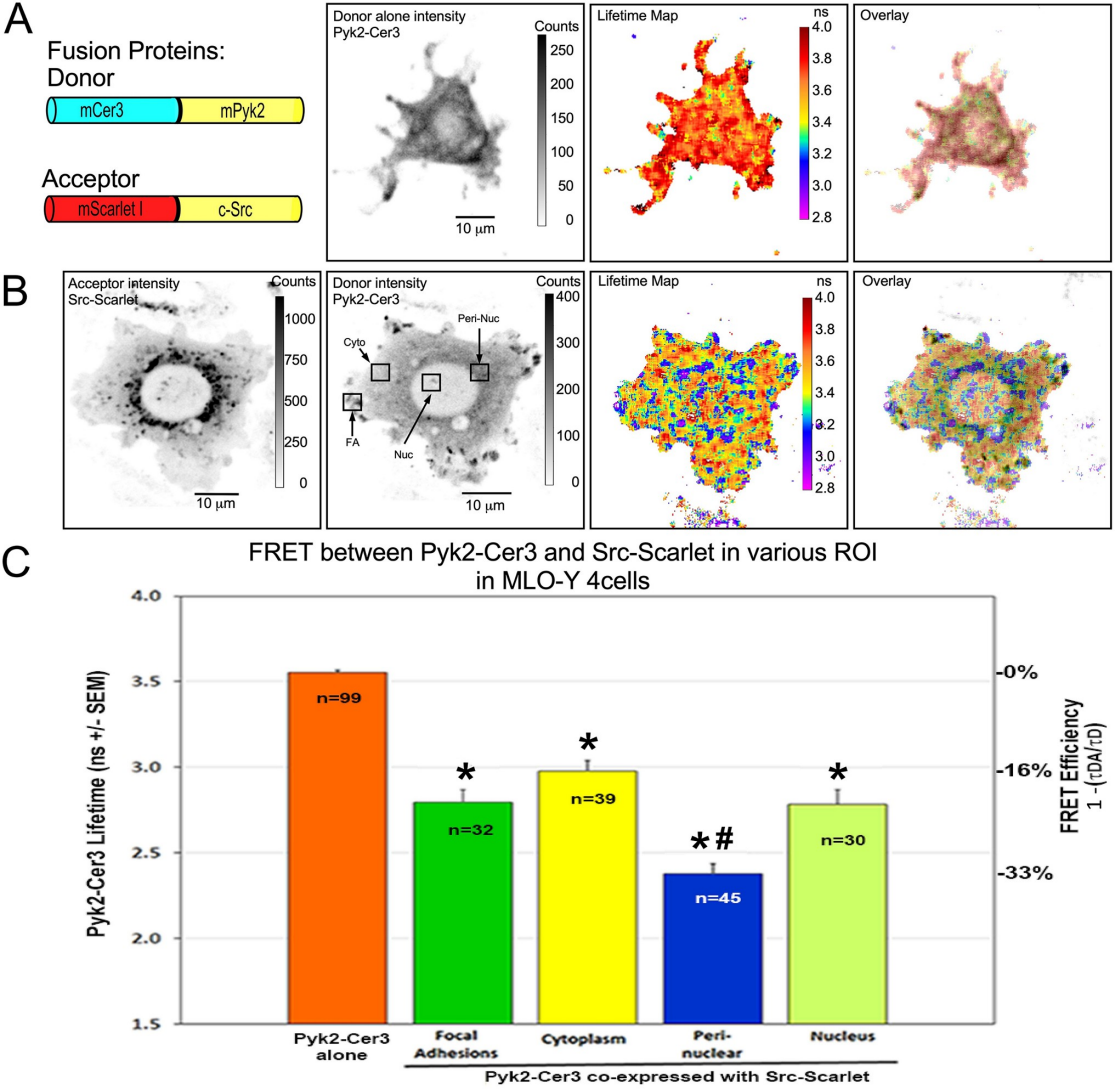
FRET between Pyk2-Cer3 and Src-Scarlet in various ROI in MLO-Y4 cells. Panel A shows (from left to right) diagrams of the Donor (Pyk2-Cer3) and Acceptor (Src-Scarlet) constructs, the intensity distribution of Pyk2-Cer3 donor expressed alone, the Lifetime map of the Pyk2-Cer3 donor alone and the IMD overlay of the Lifetime map and distribution intensity. The lookup table (LUT) indicates pixel-by-pixel lifetime with warmer colors indicating longer lifetime in nanoseconds (ns). Of note, the Lifetime map and IMD overlay in panel A demonstrate the uniform average lifetime (average of 3.56 ns) throughout the cell expressing Pyk2-Cer3 donor alone. Panel B shows cells co-transfected with (from left to right) Src-Scarlet (Acceptor) intensity, Pyk2-Cer3 (Donor) intensity, lifetime map of Pyk2-Cer3 in the presence of Src-Scarlet and the IMD overlay of the Lifetime map and distribution intensity. Here, the cooler colors in the lifetime map and IMD overlay (blues and purples) indicate shorter lifetimes consistent with where FRET is occurring between Pyk2-Cer3 and Src-Scarlet. Panel C shows quantification of the Pyk2-Cer3 Lifetimes (on the left y-axis) when Pyk2-Cer3 was expressed alone (orange bar), and in 4 regions of interest (ROI) from cells co-transfected with Pyk2-Cer3 and Src-Scarlet. Examples of where in cells the ROI were selected are illustrated in the Pyk2-Cer3 donor intensity frame of panel B, including focal adhesions (FA), cytoplasm, peri-nuclear region and inside the nucleus. FRET (on the right y-axis) between Pyk2-Cer3 and Src-Scarlet was detected in all four ROI (*p<0.05, compared to Pyk2-Cer3 alone; #p<0.05 compared to FA, cytoplasm and nucleus by one-way ANOVA on ranks followed by Dunn’s pairwise comparison) and occurred with the highest FRET efficiency in the peri-nuclear region.

Next, for FRET-FLIM studies MLO-Y4 cells were transfected with Pyk2-Cer3 alone or co-transfected with Pyk2-Cer3 and Src-Scarlet to determine if these proteins associated in a protein complex, and if so, where in cells were they interacting (Fig 2). As demonstrated by the measurements of the FRET standards (S1 Fig), the hallmark of energy transfer is the quenching of the donor lifetime, which we quantify using FLIM. When Pyk2-Cer3 was expressed alone, it was distributed diffusely throughout the cytoplasm and in focal adhesions, but was not notably concentrated in the nucleus as can be seen in both the lifetime map or, more informatively, in the Intensity Modulated Display (IMD, created by overlaying of the fluorescence lifetime map and the donor fluorescence intensity, Fig 2A). The IMD panel provides the added value of directly comparing the distribution of the fluorescence lifetimes to the actual concentration of the donor. Importantly, the lifetime of Pyk2-Cer3 was uniform throughout the cell (Fig 2A lifetime map and IMD overlay) and did not differ from the 3.6 ns lifetime of Cer3 alone (see S1 Fig). The average Pyk2-Cer3 lifetime from n = 99 regions of interest (ROI) including FA (focal adhesion), cytoplasm and nuclear regions, from 24 different cells transfected with Pyk2-Cer3 alone was 3.56 ns (Fig 2C, orange bar in panel C, n = 99 unique ROI from 24 cells). This is the lifetime that is expected for Cer3 expressed in living cells [21]. Furthermore, when Pyk2-Cer3 was expressed alone there was no significant difference in the lifetime between different ROI in cells (FA = 3.56ns +/- 0.14; Cytoplasm = 3.50ns +/-0.19; nucleus = 3.63 +/- 0.11). Strikingly, in the presence of the co-expressed Src-Scarlet, we observed significant quenching of the Pyk2-Cer3 in several different regions of the cells, and this is shown by the lifetime map and IMD overlay (Fig 2B), which is consistent with FRET between the FP tagged Pyk2 and Src. The representative cell shown in Fig 2B illustrates with black boxes the various ROI where Pyk2 and Src were localized, including focal adhesions (FA), cytoplasm, peri-nuclear and nuclear regions, and demonstrates significant quenching of the Pyk2-Cer3 in these regions. These ROI shown by the black boxes are representative of similar measurements throughout subsequent FRET-FLIM experiments. In these lifetime maps and IMD overlays, regions where quenching occurs are indicated by cooler colors (blues and purples representing shorter lifetimes) and represent locations where the FPs labeling the proteins are separated by less than approximately 80 angstroms [37]. The shorter donor lifetimes are consistent with quenching because of FRET, indicating interaction between Pyk2 and Src in these sub-cellular regions. Measurement and statistical analysis of various ROI in cells co-expressing Src-Scarlet as a FRET acceptor, the Pyk2-Cer3 lifetime was reduced in the cytoplasm to 2.98 (Fig 2C, n = 39 ROI from 22 cells). This corresponds to average FRET Efficiency (E_FRET_) of 16.2%, suggesting that Pyk2 and Src are present as an interacting complex even in regions of the cytoplasm where there is no obvious concentration of either protein (based on intensity measurements in Fig 2B). However, in the focal adhesions (FA) and the peri-nuclear regions where Src and Pyk2 appear to be concentrated, the lifetimes were reduced even further to 2.79 ns (n = 32 ROI from 22 cells), and 2.38 ns (n = 45 ROI from 22 cells), respectively (corresponding to an average E_FRET_ of 21.4% in FA and 33.2% in peri-nuclear regions) consistent with an interaction between these proteins. Interestingly, we also noted shorter lifetimes (average lifetime of 2.78 ns (n = 30 ROI from 20 cells) corresponding to E_FRET_ of 21.7%) in nuclear regions where Src and Pyk2 were far less concentrated than they were outside of the nucleus (Fig 2C). The lifetime was significantly quenched compared to that for Pyk2-Cer3 donor alone in all four ROI and highlights the fact that evaluation of lifetimes by FLIM is not affected by the level of donor and acceptor expression. FRET efficiency in the peri-nuclear region was also significantly higher than in all 3 other ROI (FA, cytoplasm and nucleus), while there was no significant difference in FRET-efficiency between FA, cytoplasm and nucleus (Fig 2C).

### Pyk2-Src interaction in focal adhesions

The mechanosome model of a cellular level mechanotransduction mechanisms predicts that detection of mechanical signals occurs by a mechanosensor located at the cell surface at sites of attachment between the cell surface and the surrounding extracellular matrix (e.g., integrins/focal adhesions). Focal adhesions are widely accepted to represent such sites at the cell surface where putative mechanosome proteins (e.g., Src and Pyk2) interact indirectly with integrin cytoplasmic domains [38, 39]; thus making focal adhesions ideally positioned to detect and respond to conformational changes in integrin subunits that occur in response to mechanical stimuli at the cell surface [40–42]. In bone the movement of interstitial fluid across the cell surface and/or mechanical strain conveyed through a deformable extracellular matrix substrate are the most significant mechanical stimuli for osteocytes [43]. Therefore, we looked in more detail at where within the focal adhesions Src and Pyk2 associated in complexes.

**Fig 3:**
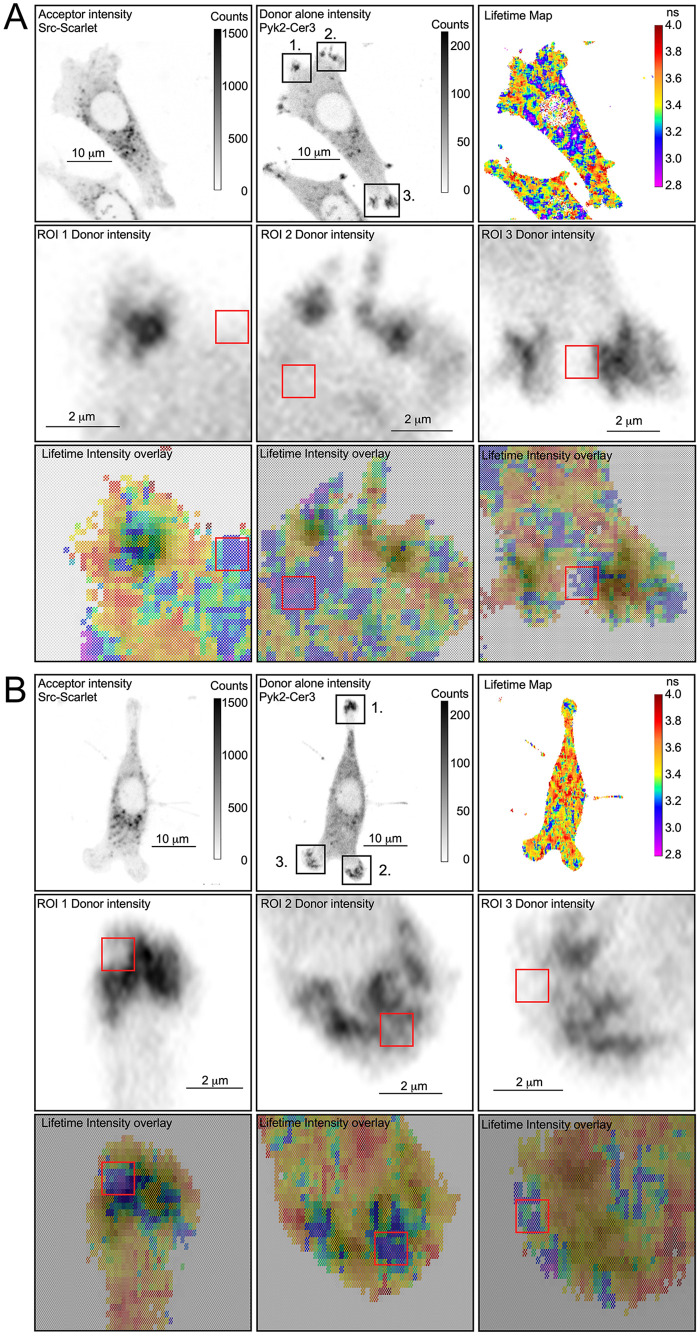
FRET between Pyk2-Cer3 and Src-Scarlet in the focal adhesions in MLO-Y4 cells. Examples of FRET between Pyk2-Cer3 and Src-Scarlet are shown from two representative cells (Panels A and B). Shown are lower and higher magnifications of three different ROI of the cell areas containing focal adhesions, designated 1, 2 and 3 in each of the two cells shown. Of note, the Lifetime maps and IMD overlay at higher magnification show in greater detail the area in and around each of the focal adhesion ROI where FRET between Pyk2-Cer3 and Src-Scarlet are occurring with the highest efficiency (cooler blue and purple colors). Red boxes are included to orient the reader to the position of highest FRET efficiency and highlight that high FRET (cooler colors) is occurring outside of the area of the FA with the highest donor intensity.

Fig 3 shows two different cells that provide examples of the quenching of Pyk2-Cer3 lifetime in the presence of Src-Scarlet in focal adhesions. As mentioned above, lifetime measurements are unaffected by variations in probe concentration. Of note, we found that the shortest Pyk2-Cer3 lifetimes were not distributed uniformly throughout the focal adhesions (indicated by cooler colors in the lifetime map and IMD overlay insets in Fig 3), and were not associated with regions where Src and Pyk2 were most concentrated (as identified by sites with the highest fluorescence intensity in insets in Fig 3). The red boxes in the insets highlight where the lifetimes are shortest (regions of highest FRET). Critically, the IMD demonstrated that the donor intensity is lower in these regions of high FRET that were notably outside of the regions where donor intensity is highest (which by definition indicates the position of the focal adhesion as indicated by, in this case, Pyk2-Cer3). Rather, the most quenched lifetimes, and therefore the most efficient FRET between Src and Pyk2 were often located nearer the periphery of the focal adhesions, at least as identified by the distribution of total Src and Pyk2 in the adhesions (compare regions in and around the red boxes in the donor intensity panel and the IMD overlay of the lifetime in Fig 3).

#### FRET-FLIM analysis of MBD2 interaction between Src and Pyk2

**Fig 4:**
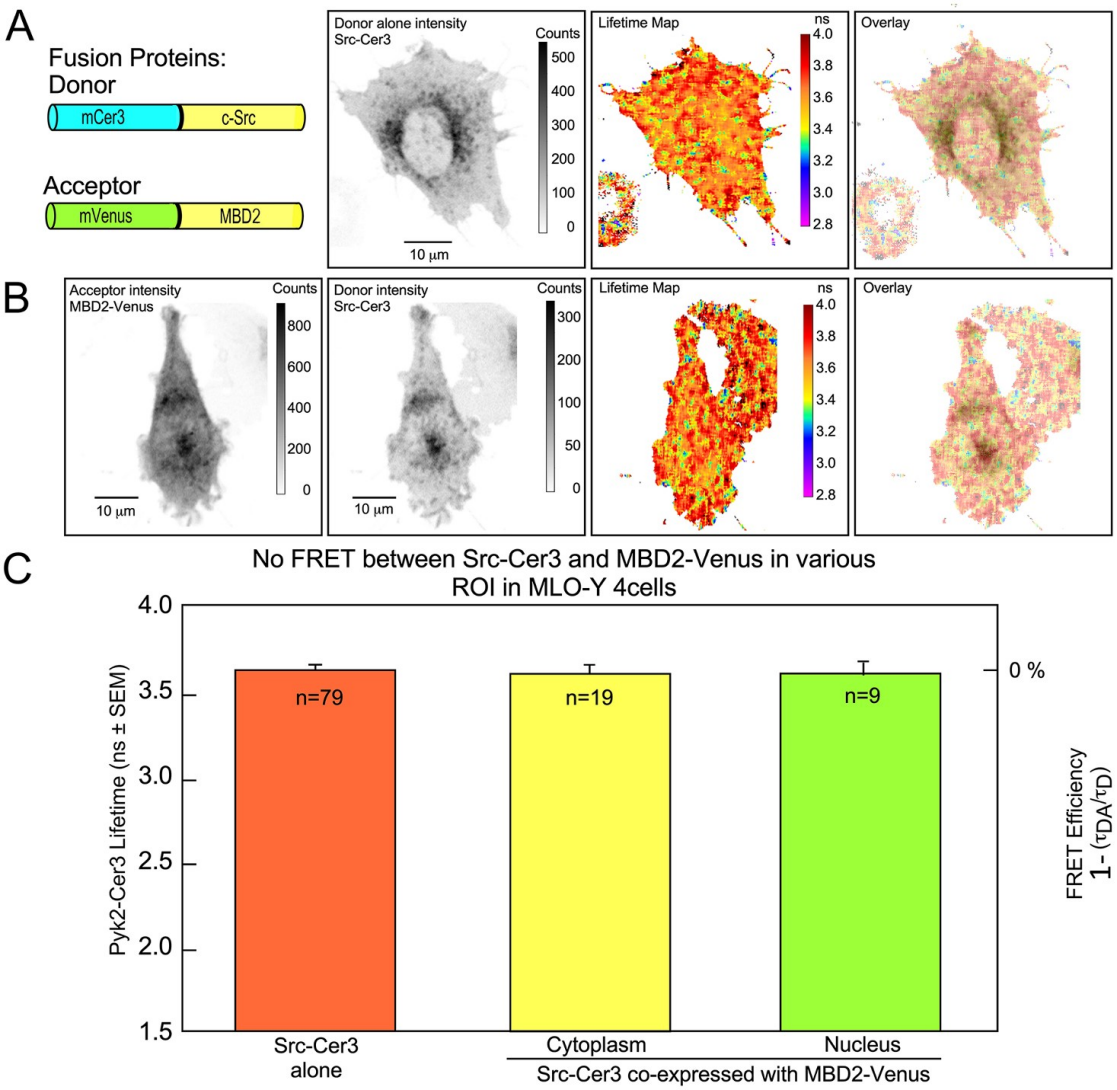
FRET was not detected between Src-Cer3 and MBD2-Venus in MLO-Y4 cells. Panel A shows (from left to right) diagrams of the Donor (Src-Cer3) and Acceptor (MBD2-Venus) constructs (left side), and the distribution intensity of Src-Cer3 donor expressed alone, the Lifetime map of the Src-Cer3 donor alone and the IMD overlay of the Lifetime map and donor intensity. Of note, the Lifetime map and IMD overlay in panel A demonstrates the uniform lifetime (average of 3.61 ns) throughout the cell transfected with Src-Cer3 donor alone. Panel B shows (from left to right) cells co-transfected with MBD2-Venus (Acceptor), Src-Cer3 (Donor), the Lifetime map of Src-Cer3 in the presence of MBD2-Venus, and the IMD overlay of the Lifetime map and donor intensity. Of note, no cooler colors (blues and purples) are seen indicating that FRET is not occurring between Src-Cer3 and MBD2-Venus. Panel C shows quantification of the Src-Cer3 Lifetimes (on the left y-axis) when Src-Cer3 was expressed alone (orange bar), and in 2 regions of interest (ROI) from cells co-transfected with Src-Cer3 and MBD2-Venus (cytoplasm and nucleus). FRET efficiency is zero throughout the cell, regardless of differences in the sub-cellular concentration of each protein (calculated as 1 minus the ratio of lifetime of the donor/acceptor (τ_DA_) to the donor alone (τ_D_) and indicated on the right y-axis).

**Fig 5:**
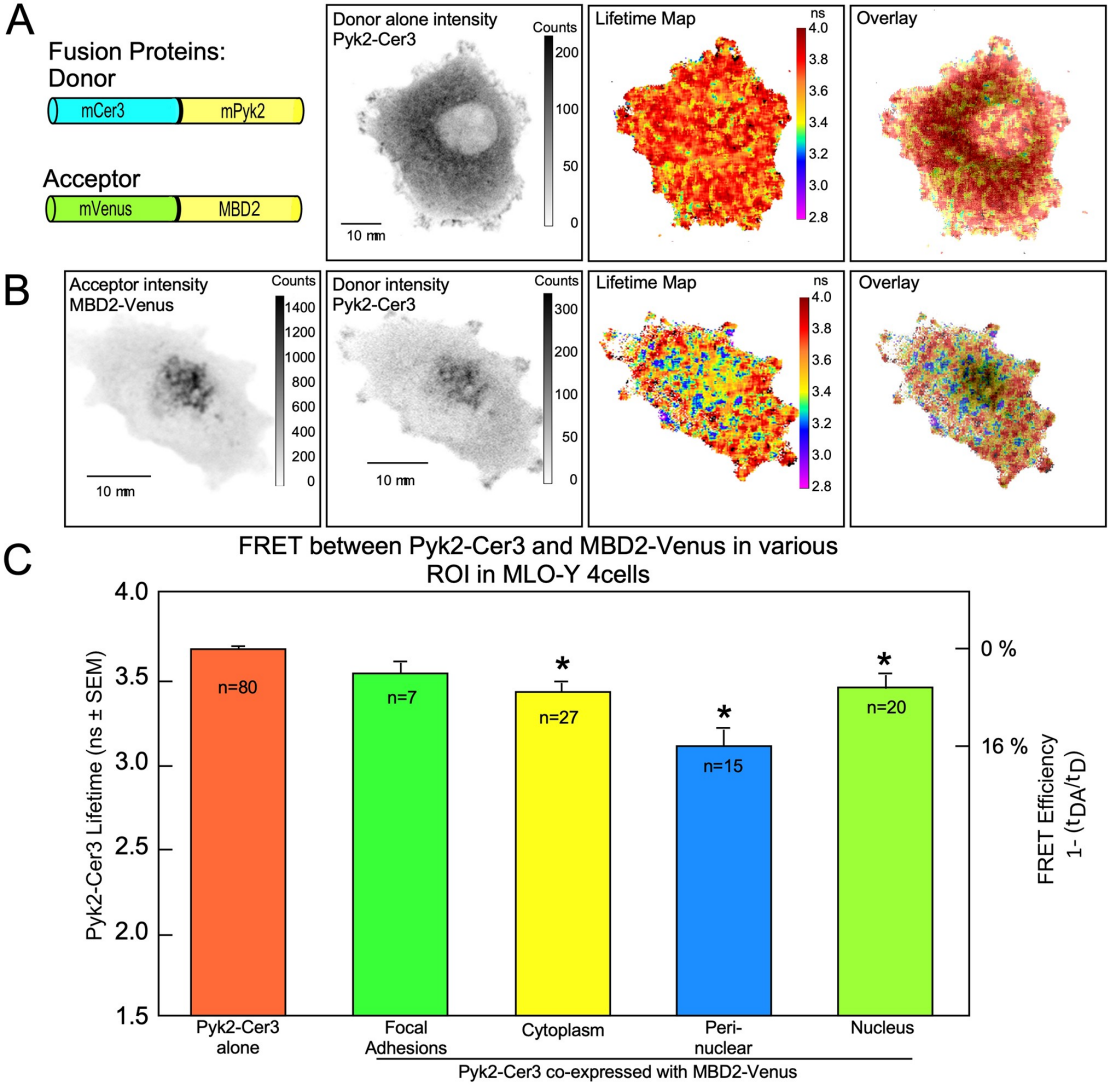
FRET between Pyk2-Cer3 and MBD2-Venus in various ROI in MLO-Y4 cells. Panel A shows (from left to right) diagrams of the Donor (Pyk2-Cer3) and Acceptor (MBD2-Venus) constructs, the distribution intensity of Pyk2-Cer3 donor expressed alone, the Lifetime map of the Pyk2-Cer3 donor alone, and the IMD overlay of the lifetime map and donor intensity. Of note, the Lifetime map and IMD overlay in panel A demonstrate the uniform lifetime (average of 3.68 ns) throughout the cell transfected with Pyk2-Cer3 donor alone. Panel B shows (from left to right) cells co-transfected with MBD2-Venus (Acceptor), Pyk2-Cer3 (Donor), the Lifetime map of Pyk2-Cer3 in the presence of MBD2-Venus, and the IMD overlay of the Lifetime map and donor intensity. Of note, the cooler colors (blues and purples) indicate where FRET is occurring between Pyk2-Cer3 and MBD2-Venus. Panel C shows quantification of the Pyk2-Cer3 Lifetimes (on the left y-axis) when Pyk2-Cer3 was expressed alone (orange bar), and in 4 regions of interest (ROI) from cells co-transfected with Pyk2-Cer3 and MBD2-Venus. FRET between Pyk2-Cer3 and MBD2-Venus was detected in the cytoplasm, peri-nuclear area and nucleus (*p<0.05, compared to Pyk2-Cer3 alone; #p<0.05 compared to focal adhesions, cytoplasm and nucleus, by one-way ANOVA followed by Dunn’s pairwise comparison) and occurred with the highest FRET efficiency in the peri-nuclear region (calculated as 1 minus the ratio of lifetime of the donor/acceptor (τ_DA_) to the donor alone (τ_D_) and indicated on the right y-axis).

We next used the FRET-FLIM approach to determine whether MBD2 interacted with either Src or Pyk2. MLO-Y4 cells were co-transfected with MBD2-Venus along with either Src-Cer3 (Fig 4) or Pyk2-Cer3 (Fig 5). In these experiments, MBD2-Venus served as the acceptor fluorophore for the donor fluorophore -Cerulean3 fused to either Pyk2 or Src. When Src-Cer3 was co-transfected with MBD2-Venus we observed that there was no quenching of the donor (Src-Cer3) lifetime (compare lifetime maps/IMD overlay Fig 4A and 4B). The lifetime of Src-Cer3 in the presence of MBD2-Venus was indistinguishable from the lifetime of Src-Cer3 alone (Fig 4C). Quantification of ROI in the cytoplasm and nucleus further confirmed that Src-Cer3 lifetime was not different (E_FRET_ = 0%) when cells were co-transfected with Src-Cer3 and MBD2-Venus (Fig 3C).

We next examined whether Pyk2 interacted with MBD2 and found that the lifetime of Pyk2-Cer3 was quenched in the presence of MBD2-Venus (Fig 5B, lifetime map/IMD overlay). In cells expressing Pyk2-Cer3 alone the average (unquenched) lifetime was 3.56 ns (orange bar in Fig 5C) consistent with the results shown in Fig 2 for Pyk2-Cer3 alone. When co-transfected with MBD2-Venus as an acceptor, Pyk2-Cer3 lifetimes in three ROI analyzed (cytoplasm, peri-nuclear region and nucleus) were significantly quenched suggesting that Pyk2-Cer3 and MBD2-Venus were associated in these sub-cellular regions. Interestingly, lifetimes in the peri-nuclear region were the shortest of the four ROI we analyzed and were significantly shorter than any other ROI. The shortest lifetime in perinuclear regions is normally an indication of stronger interaction between Pyk2 and MBD2 in this area. However, several alternative possibilities could account for this difference, including the speed of folding of the FP, variation in the concentration of FP in distinct regions of the cell, unique microenvironments around FPs in different regions of the cells as well as the potential for different conformation of FP protein complexes in this region.

**Fig 6:**
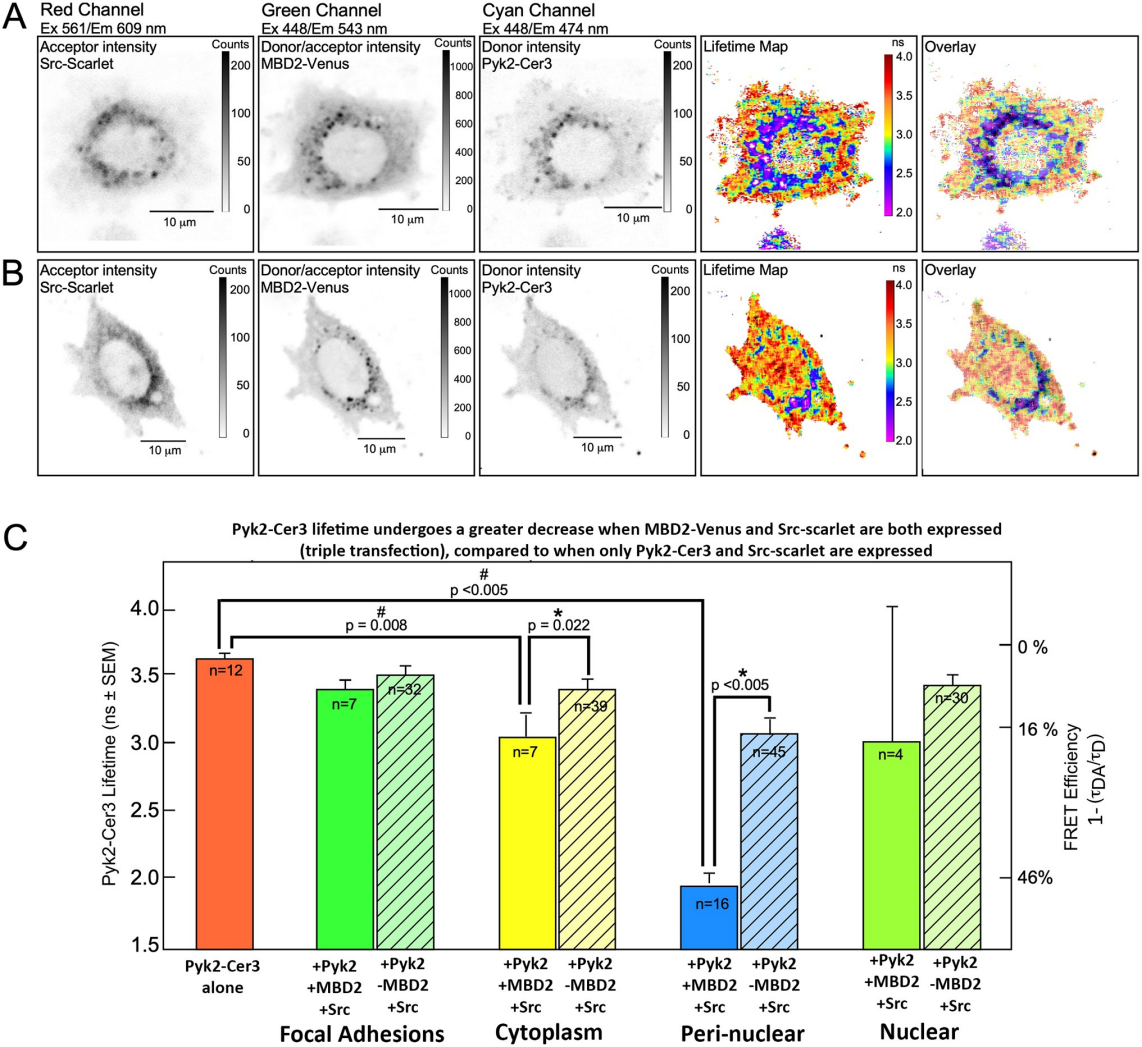
FRET between Pyk2-Cer3 and MBD2-Venus is altered when cells are triple transfected, particularly in the peri-nuclear region in MLO-Y4 cells. Panels A and B show two different representative cells that were triple-transfected with Src-Scarlet (acceptor), MBD2-Venus (donor or acceptor) and Pyk2-Cer3 (donor), as indicated above the rows. Lifetime maps and IMD overlays of the Lifetime map and donor intensity of Pyk2-Cer3 are shown on the rightmost two frames of each row. The graph below (Panel C) shows quantification of Pyk2-Cer3 lifetime in cells transfected with Pyk2-Cer3 alone (average of 3.65 ns, orange bar on the far left side) and in four ROI from cells co-expressing Pyk2, MBD2 and Src, or expressing only Pyk2 and Src (without MBD2) as indicated along the x-axis. Note that the data from the striped bars is obtained from the experiments shown in Fig 2. The Pyk2-Cer3 lifetime in the peri-nuclear region and cytoplasm ROI in cells expressing MBD2 (solid bars) was significantly shorter compared to cells not expressing MBD2 (striped bar). The Pyk2-Cer3 lifetimes were also significantly shorter in the peri-nuclear and cytoplasm ROI in cells expressing MBD2 compared Pyk2-Cer3 alone (*p values are as indicated between the groups highlighted).

Together our observations show that FP-Src was typically concentrated in the peri-nuclear region, as well as in focal adhesions (S2 Fig, Figs 2 and 3). We demonstrated by FRET-FLIM that Src and Pyk2 appear to associate in both the peri-nuclear regions and focal adhesions. In stark contrast, we did not detect an association between Src and MBD2 in these same regions (Fig 4). However, our FLIM results do suggest that Pyk2 and MBD2 associate in the peri-nuclear regions (Fig 5B, lifetime map/IMD overlay). Collectively these results support the existence of a Pyk2-containing protein complex that may possibly function as a mechanosome involving Src and MBD in the peri-nuclear region but begs the question of how these proteins are interacting. To begin to address this question, we performed a triple-transfection of Src-Scarlet, Pyk2-Cer3 and MBD2-Venus in the same living cells (Fig 6). Here, Scarlet can serve as a FRET acceptor for both Cer3 and Venus, while Venus serves as an acceptor for Cer3 and a donor for Scarlet. In support of the earlier observation (Fig 4), we did not observe quenching of MBD2-Venus in the presence of Src-Scarlet (MBD2-Venus alone lifetime = 3.60 +/- 0.15ns; MBD2-Venus/Src-Scarlet = 3.59 +/- 0.136 ns in the Cytoplasm; MBD2-Venus alone lifetime = 3.64 +/- 0.125 ns; MBD2-Venuse/Src-Scarlet lifetime = 3.61 +/- 0.10 ns in the Nucleus). Strikingly however, compared to cells not expressing MBD2-Venus we observed a further quenching of Pyk2-Cer3 lifetime in the peri-nuclear region in cells when both Src-Scarlet and MBD2-Venus were present (lifetime maps and IMD overlay in Fig 6A and 6B, showing 2 different cells). In this experiment both Venus and Scarlet can potentially act as acceptor fluorophores for Cer3, in contrast to cells co-transfected with Pyk2-Cer3 and Src-Scarlet (Fig 2). Panel C in Fig 6 compares the data for this triple-transfection experiment to the data shown in Fig 2 in which Pyk2-Cer3 and Src-Scarlet were co-transfected (but without MBD2-Venus, represented by the hatched bars in Fig 6C). We would expect that the additive effect of two FRET acceptors on the fluorescence lifetime of the FRET donor means that both interactions are present in the same pixel area. However, an alternate possible interpretations of this result could indicate that the effect of triple transfection, including MBD2-Venus is having a measurable impact on the conformation of a Src-Pyk2-MBD2 complex in the peri-nuclear region of the cell potentially providing an additive effect on quenching of Pyk2-Cer3 lifetime over the quenching effect MBD2-Venus alone.

### Effects of fluid flow on mechanosome intracellular distribution

**Fig 7:**
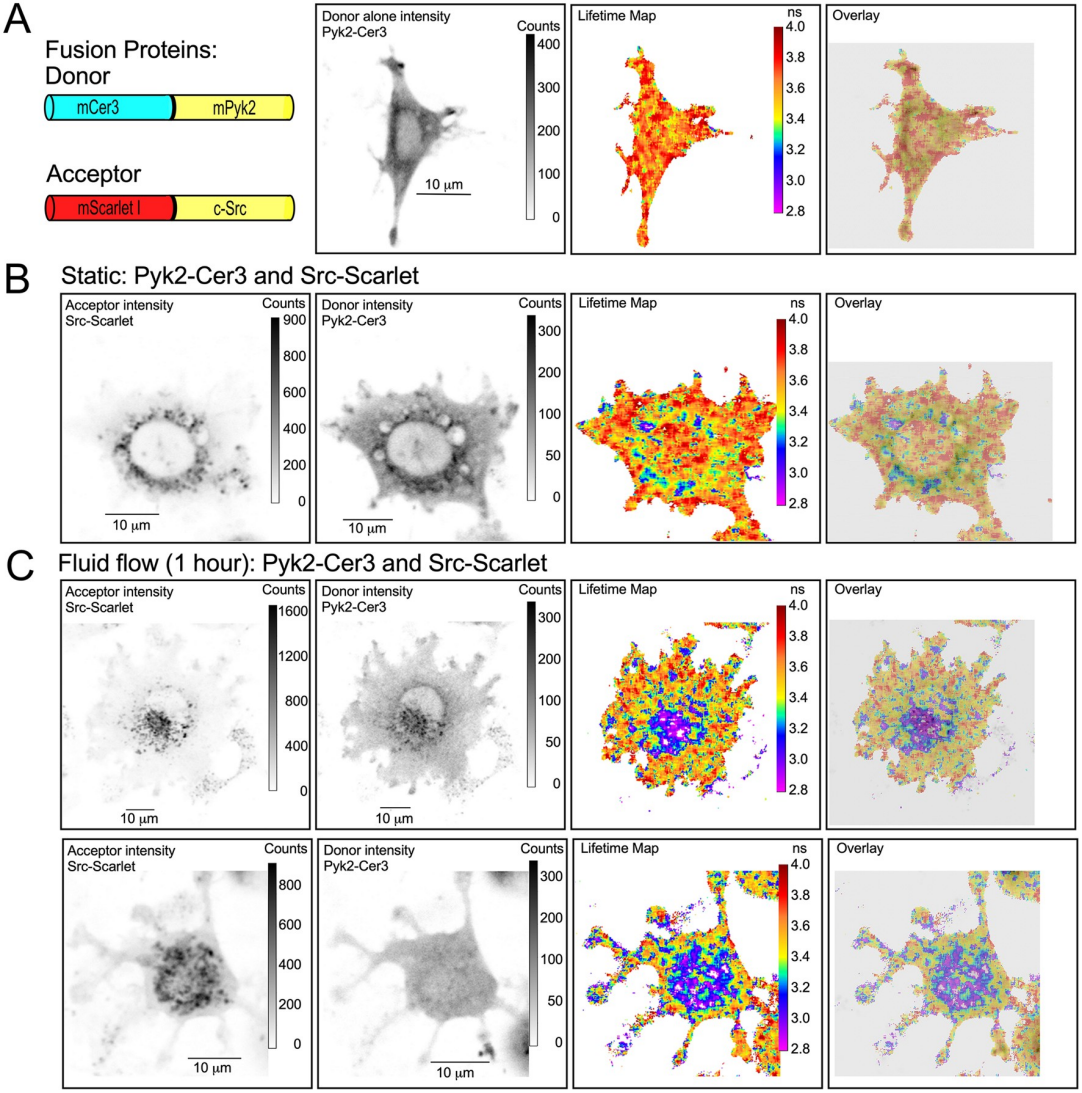
Application of a mechanical stimulus (fluid flow) induces peri-nuclear/nuclear redistribution of Pyk2-Cer3/Src-Scarlet interacting complexes in MLO-Y4 cells. Panel A shows (from left to right) a schematic of the probes used in this experiment, a donor intensity image of a representative cell transfected with the Pyk2-Cer3 (donor) alone, the Lifetime map and IMD overlay of the Lifetime map and donor intensity image. As shown in previous figures, nearly all the Pyk2-Cer3 lifetimes are in the unquenched range of approximately 3.6 ns. Panel B shows (from left to right) a cell co-transfected with Src-Scarlet (acceptor), Pyk2-Cer3 (donor), the lifetime map, and the IMD overlay of the Lifetime map and donor intensity. Note that as shown in Fig 2, shorter lifetimes indicating FRET between Pyk2-Cer3 and Src-Scarlet are found most prominently in the peri-nuclear region and in the focal adhesions. The cell in panel B was maintained in static culture with an effort to minimize fluid movement as in all other experiments described. In contrast, the two representative cells shown in in the upper and lower rows of panel C that were also co-transfected with Src-Scarlet and Pyk2-Cer3 were subjected to one hour of fluid flow by placing the culture dish on an orbital shaker. As seen in the lifetime maps and IMD overlays of the lifetime map and donor intensity the shortest lifetimes (blue/purple colors) appeared much more coalesced in the middle of the cell in the peri-nuclear and nuclear region.

Finally, we performed a preliminary experiment looking at whether these proteins behave in a manner consistent with our models of mechanosome function in which we posit that mechanical stimulation of cells should stimulate activation and intracellular movement of mechanosome complexes. To do so, we applied a crude mechanical stimulus to MLO-Y4 cells co-transfected with Pyk2-Cer3 and Src-scarlet by generating fluid movement over the cultured cells using cells in microscopy chambers directly on an orbital shaking platform for one hour (Fig 7). Our goal was to determine the impact of mechanical stimulation of Pyk2-Src protein complexes. We estimate that, compared to cells maintained in static culture (Fig 7) a subset of approximately 10–20% of cells subjected to fluid movement (Fig 7C, showing 2 representative cells) responded by redistributing Pyk2-Cer3/Src-Scarlet complexes with reduced lifetimes toward the cell interior and coalesced in the peri-nuclear region and apparently within the cell nucleus. We recognize that using an orbital shaker is a crude method for mechanically stimulating the cells, however it was the only option available using or FRET-FLIM compatible cell chambers. Nonetheless, this observation is consistent with the hypothesis that Pyk2 and Src physically interact to form part of a multi-protein mechanosome complex in osteocytes that can redistribute from focal adhesions and cytoplasm around the cell periphery to nuclear and peri-nuclear regions of the cell in response to mechanical stimulation.

## Discussion

We describe here for the first time direct imaging of a multi-protein complex in living cells that possess the key characteristics of a functional mechanosome as originally hypothesized in 2003 [3]. The generation of fluorescent tagged mechanosome proteins combined with the spatial resolution afforded by the imaging technique of FRET-FLIM allowed us to show that a protein complex containing Pyk2 and Src, possible involving MBD2 can be imaged in MLO-Y4 osteocytes. While we detected this complex diffusely throughout the cytoplasm, we most notably observed that the complex was concentrated in the region surrounding the nucleus (peri-nuclear) as well as in the nucleus. Src and Pyk2 were abundant in focal adhesions and interacted as evidenced by the strong quenching of the FP-Pyk2 lifetime by FP-Src measured by FRET-FLIM. This finding of an interaction between Src and Pyk2 in focal adhesions is of particular significance because of the key role attributed to mechanosome proteins at these sites of interaction between integrins and the ECM (extracellular matrix). As prime candidates for important sites of mechanosensation, integrin-containing focal adhesions are the most likely cellular structures from which a multi protein mechanosome complex could be launched in response to detection of a mechanical stimulus.

Our data suggest, although cannot prove, that Pyk2 is associated with both Src and MBD2 in the peri-nuclear region of the cell. Interestingly, we did not detect an association between Src and MBD2 in these regions, but when all three proteins were present in the peri-nuclear region, we did observe further quenching of the FP-Pyk2. A potential interpretation of these observation is that Src/Pyk2 leaves the focal adhesions in response to mechanical stimulation and that Pyk2 becomes physically associated with MBD2 in the peri-nuclear region. It is possible that the Src/Pyk2/MBD2 mechanosome complex exists in a unique conformation in this region surrounding the nucleus. As with all studies that utilize overexpression of a cellular proteins, caution must be used with interpreting results since higher than endogenous levels of expressed protein could impact the normal biology of the proteins. To minimize the potential confounding effects of FP-Src, FP-Pyk2 and FP-MBD2 overexpression in these studies we selected cells for FRET-FLIM analysis that maintained a well spread morphology typical for MLO-Y4 cells and did not select cells for analysis that appeared overly bright, which could indicate extreme overexpression of the exogenous protein. When we estimated the level of exogenous protein expression relative to the endogenous protein in the total population of cells using immunoblot analysis of cells transfected with a single construct, we found that FP-Src and FP-Pyk2 were each overexpressed by approximately 2–4 fold, with little variation between transfections (n = 3 in multiple trials, Fig 1A and 1B). In contrast, expression of FP-MBD2 appeared to be much lower based on our inability to detect FP-MBD2 using any of four different MBD2 antibodies that detected endogenous MBD2. We were however able to confirm expression of FP-MBD2 using an anti-GFP antibody that recognized the FP (Venus) portion of the fusion protein at the approximate molecular weight expected for MBD2-Venus (Fig 1C). As expected, we saw a wide range of expression levels among cells within each population of transfected cells. In addition, a noted advantage of FLIM is that the results are independent of the level of expression of either donor-acceptor pair.

The technique of FRET-FLIM is the only approach we are aware of that would be suitable for detecting interaction between these putative mechanosome proteins and analysis of their sub-cellular distribution within living cells at this level of sub-cellular resolution. The time required for making FLIM measurements in live cells is approximately 30 seconds, which places limits on the temporal resolution of very rapid, dynamic events within cells. Thus, it would be very difficult to follow and measure the association of protein complexes and their movements within the same cell over time following mechanical stimulation. Definitive proof of our model can come only from following the movement of the mechanosome in an individual triple-transfected cell over time following mechanical stimulation. Such an experiment will be technically quite challenging but will be justified in future studies based on the observations we report here. Potentially this will be possible by following a single cell in a flow chamber. We are pursuing this approach and hope to be able to describe in future studies the interaction and movement of mechanosomes before, during and after application of fluid flow through the flow chamber.

Limitations of this methodology include not only the technical difficulty of following protein complexes in the same cell over time mentioned above, but also the need to modify putative mechanosome proteins with fluorescent probes suitable for analysis by FRET-FLIM. As such, one must consider the confounding risk that genetically modifying the endogenous protein may simultaneously alter conformation and behavior of the protein, including how modified proteins interact with each other. It is also difficult to assess the impact of adding a fluorescent protein tag on the initial intracellular distribution (localization) pattern of proteins in cells. For the proteins examined in this study, the FP-tagged versions of Src, Pyk2 and MBD2 are localized in cells following transfection in a pattern that is indistinguishable from that of the endogenous proteins detected by immunostaining [44–47]. This gives us some confidence that the addition of the FP tag is not impacting where in cells the expressed proteins are localized. As in all studies of protein expression in cells (with or without FP tag), potential artifacts of over-expression are a concern. In our results, the lack of interaction between MBD2 and Src (as assessed by FRET-FLIM) argue against protein over-expression generally inducing interactions between two proteins that might not otherwise be occurring between endogenous proteins. Also, these studies were, in part, undertaken based on our previous observation of a Pyk2-containing protein complex that possibly interacts with Src and MBD2 that was suggested, but not confirmed by immunoprecipitation from lysed cells [6]. Therefore, the FRET-FLIM studies reported here can be viewed as supporting earlier co-immunoprecipitation results suggesting the existence of a multi-protein interacting complex.

Our observation that the Src/Pyk2/MBD2 mechanosome was frequently seen concentrated in the peri-nuclear region of the cell is consistent with the notion that mechanosome complexes were predicted to accumulate around the nucleus prior to migrating inside the nucleus where they can bind to DNA and regulate protein levels by enhancing or inhibiting expression of specific genes. To explore this possibility further, we subjected cells co-transfected with Pyk2-Cer3 and Src-Scarlet to mechanical stimulation by fluid flow using an orbital shaker prior to imaging by FRET-FLIM. While an orbital shaker is not an ideal method for applying fluid shear stress to cells, the chambers we used for FRET-FLIM did not allow any other method, such as application of a defined oscillatory flow profile with a pump system that our group used previously [48–51]. Nevertheless, oscillating flow is approximated by orbital shaking [42, 52, 53] and an oscillating flow profile most closely approximates fluid movement in the canalicular system in bone [54, 55]. We found that in a subset of cells (qualitatively estimated to be between 10–20% of the cells analyzed) there was a change in the distribution of interacting complexes such that the majority of quenched Pyk2-Cer3 lifetimes were seen in and around the nucleus (see Fig 7 for representative examples). Although it is unclear why such a redistribution of interacting Src/Pyk2 complexes was seen in only a minority of cells, this result was notable because comparable peri-nuclear/nuclear accumulation of Src and Pyk2 were not typically observed in cells not subjected to mechanical stimulation by fluid flow. It must be noted however that routine handling of cells for all these studies unavoidably generated some fluid movement within the chambers as cells were moved on to the microscope stage. It is possible the levels of peri-nuclear and nuclear localization of quenched molecules that we considered to be typical in our (non-fluid flow) studies were affected by that incidental fluid movement. This possibility is difficult to test since generation of even some minimal fluid movement during handling cannot be avoided. A priority for our future studies is development of an experimental system compatible with live cell FRET-FLIM imaging over time while applying oscillating fluid flow to cells on the inverted microscope stage.

In summary, our results are consistent with the hypothesis first put forward in 2003 [3–5] that cells may respond to mechanical stimulation by activating multi-protein signaling complexes (mechanosomes). These mechanosomes may be released (or “launched”) from their associations with the cytoplasmic domains of cell surface mechanosensors (integrins) and transmit information to the nucleus by physically translocating first to the regions around the nucleus in preparation for eventual passage into the nucleus where they can regulate gene expression. Clearly other factors could be and likely are involved in driving the intracellular movement of multi-protein complexes, such as the complex we report here, and conclusions about the precise mechanisms responsible for directional movement and targeting of mechanosome complexes to the nuclear region should be made with caution. For example, fluid shear stress promotes dramatic reorganization of the cytoskeleton in bone cells as we reported over 20 years ago [1, 42] and stimulates mobilization of intracellular calcium that is associated with fluid flow-induced changes in gene expression, which, in at least some cases, is dependent upon calcium availability [56, 57]. Any of these processes could affect intracellular movement of protein complexes. In that regard, it will be particularly interesting to determine if, for example, agents that inhibit cytoskeletal filament systems, organelle trafficking, calcium mobilization or integrin-mediated adhesions to the extracellular matrix impact fluid flow-induced reorganization of these putative mechanosomes.

## Supporting information

S1 Fig Construction and characterization of FRET standards. The FRET standards were produced using either mCer3, mVenus, or mScarlet. The donor fluorophore mCer3 was directly coupled to either the acceptors mVenus or mScarlet, and the donor mVenus was coupled directly to the acceptor mScarlet through a 10 amino acid (aa) linker as described earlier [25]. The FRET standards were used to verify the FRET-FLIM measurements made using these monomeric FPs coupled to Src, Pyk2 or MBD2. The measured lifetimes of the either Donor alone (Cer3 or Venus) are shown along with the quenched lifetime of the three FRET standard constructs (FRET 10 bars) as indicated. FRET efficiencies (E_FRET_) for each Donor alone and FRET 10 pair are shown above each pair of bars.
(TIF)

S2 Fig Sub-cellular localization of putative mechanosome protein FP constructs in MLO-Y4 cells. Three representative examples each of the distribution of MBD2-Venus, Pyk2-Cer3 or Src-Cer3 are shown. Each construct was expressed alone, the lookup table (LUT) indicates intensity and the scale bar is 10 μm in length. As expected, MBD2-Venus localized most prominently in the nucleus. Pyk2-Cer3 and Src-Cer3 each localized in the focal adhesions (arrowheads) and in the peri-nuclear region. Localization of Src-Scarlet (not shown) was indistinguishable from that of Src-Cer3. Peri-nuclear localization was especially prominent in the Src-FP constructs. All constructs were present diffusely throughout the cytoplasm, while Src and Pyk2 were notably less abundant in the nucleus.
(TIF)

S1 Raw images (TIF)
