# Supplementary figures and images for: Direct visualization by FRET-FLIM of a putative mechanosome complex involving Src, Pyk2 and MBD2 in living MLO-Y4 cells
Source: PLoS One. 2021 Dec 23;16(12):e0261660. doi: 10.1371/journal.pone.0261660 (PMC8699642; doi:10.1371/journal.pone.0261660)

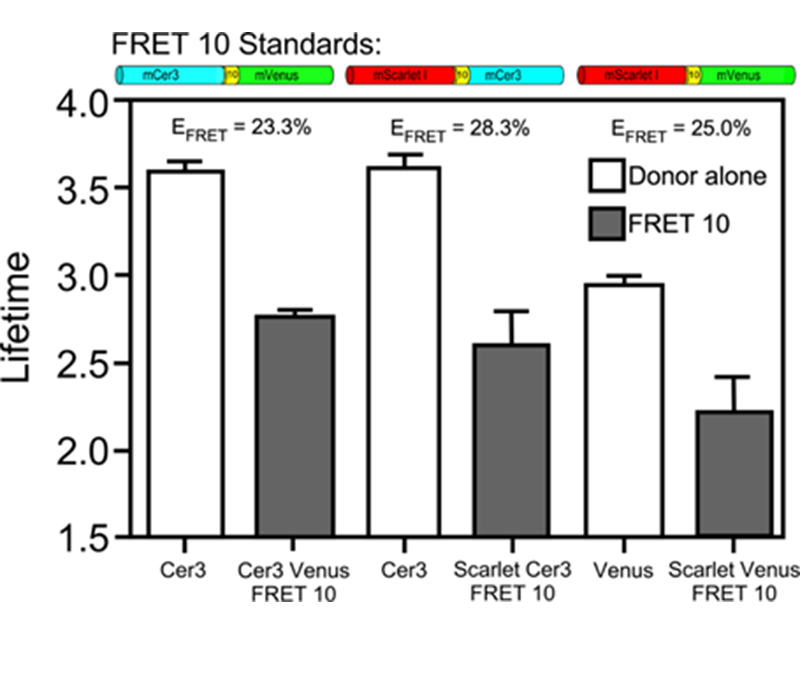

Supplement: S1 Fig — The FRET standards were produced using either mCer3, mVenus, or mScarlet. The donor fluorophore mCer3 was directly coupled to either the acceptors mVenus or mScarlet, and the donor mVenus was coupled directly to the acceptor mScarlet through a 10 amino acid (aa) linker as described earlier [25]. The FRET standards were used to verify the FRET-FLIM measurements made using these monomeric FPs coupled to Src, Pyk2 or MBD2. The measured lifetimes of the either Donor alone (Cer3 or Venus) are shown along with the quenched lifetime of the three FRET standard constructs (FRET 10 bars) as indicated. FRET efficiencies (EFRET) for each Donor alone and FRET 10 pair are shown above each pair of bars. (TIF) [file pone.0261660.s001.tif]

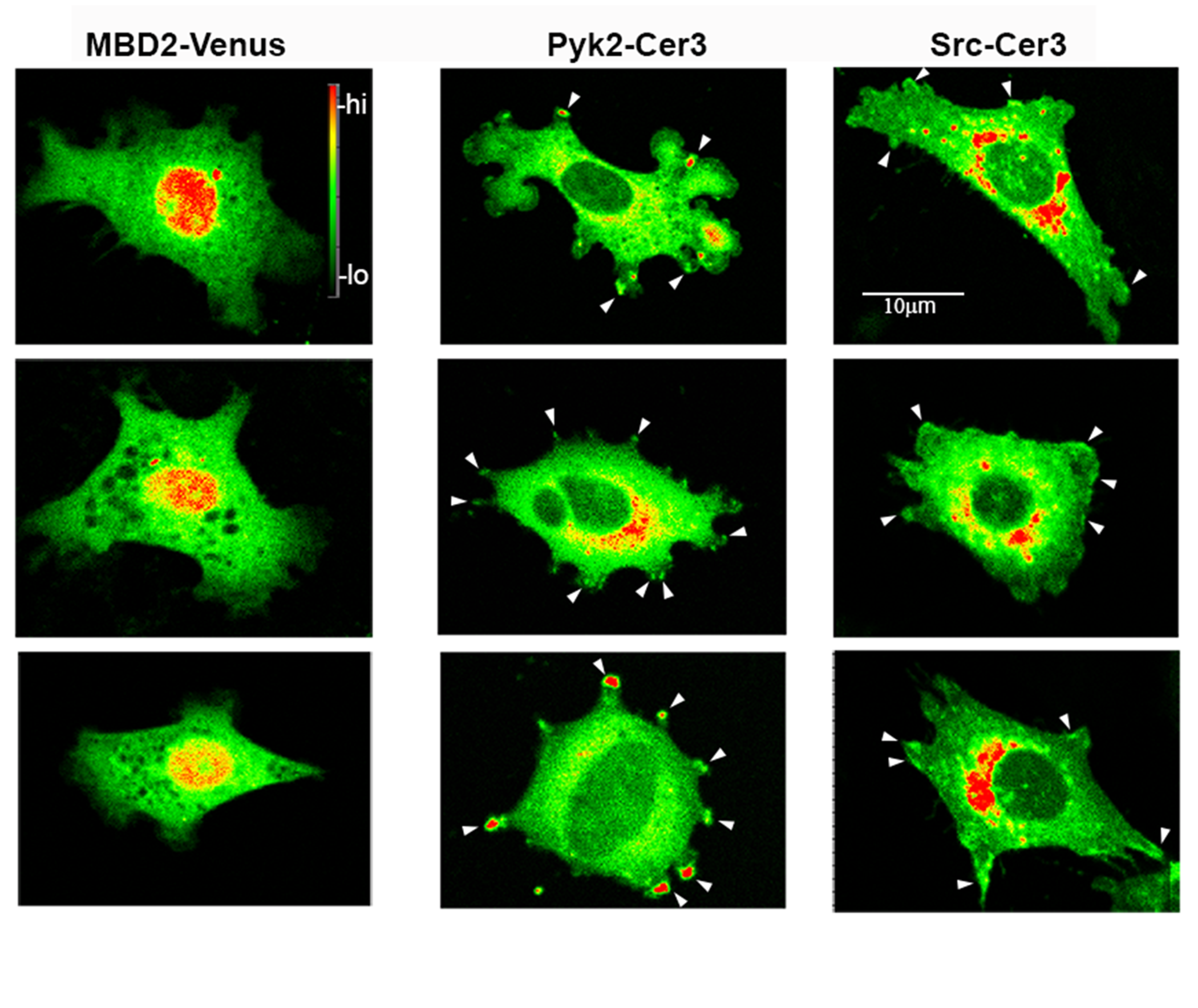

Supplement: S2 Fig — Three representative examples each of the distribution of MBD2-Venus, Pyk2-Cer3 or Src-Cer3 are shown. Each construct was expressed alone, the lookup table (LUT) indicates intensity and the scale bar is 10 μm in length. As expected, MBD2-Venus localized most prominently in the nucleus. Pyk2-Cer3 and Src-Cer3 each localized in the focal adhesions (arrowheads) and in the peri-nuclear region. Localization of Src-Scarlet (not shown) was indistinguishable from that of Src-Cer3. Peri-nuclear localization was especially prominent in the Src-FP constructs. All constructs were present diffusely throughout the cytoplasm, while Src and Pyk2 were notably less abundant in the nucleus. (TIF) [file pone.0261660.s002.tif]

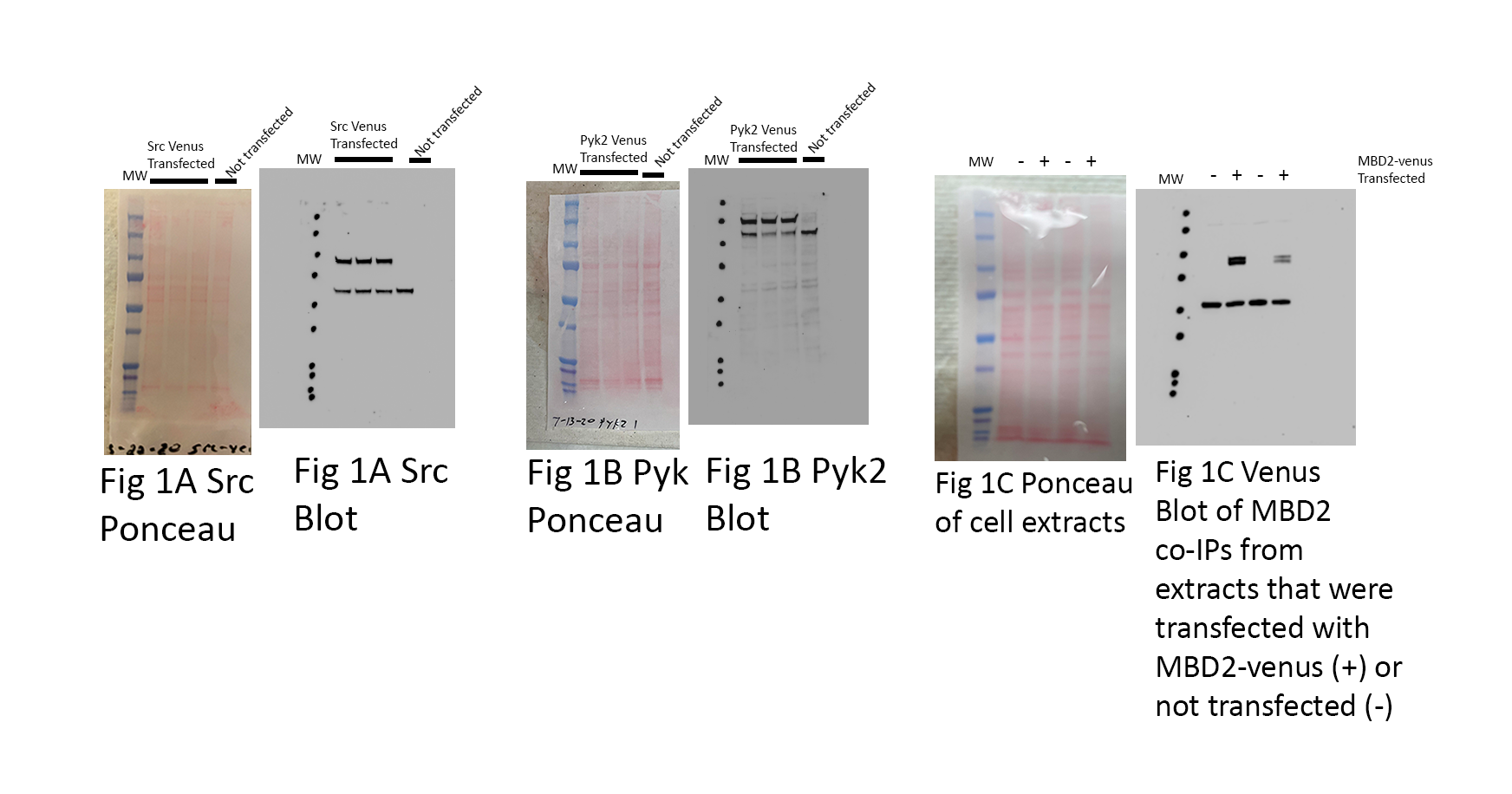

Supplement: S1 Raw images — (TIF) [file pone.0261660.s003.tif]
